# Epidemiological study of relapsing fever borreliae detected in *Haemaphysalis* ticks and wild animals in the western part of Japan

**DOI:** 10.1371/journal.pone.0174727

**Published:** 2017-03-31

**Authors:** Kiwa Furuno, Kyunglee Lee, Yukie Itoh, Kazuo Suzuki, Kenzo Yonemitsu, Ryusei Kuwata, Hiroshi Shimoda, Masahisa Watarai, Ken Maeda, Ai Takano

**Affiliations:** 1 Department of Veterinary Medicine, Joint Faculty of Veterinary Medicine, Yamaguchi University, Yamaguchi, Yamaguchi, Japan; 2 Cetacean research institute, National Institute of Fisheries Science, Nam-gu, Ulsan, Republic of Korea; 3 Hikiiwa Park Center, Tanabe, Wakayama, Japan; 4 The United Graduate School of Veterinary Science, Yamaguchi University, Yamaguchi, Yamaguchi, Japan; University of Kentucky College of Medicine, UNITED STATES

## Abstract

The genus *Borrelia* comprises arthropod-borne bacteria, which are infectious agents in vertebrates. They are mainly transmitted by ixodid or argasid ticks. In Hokkaido, Japan, *Borrelia* spp. were found in deer and *Haemaphysalis* ticks between 2011 and 2013; however, the study was limited to a particular area. Therefore, in the present study, we conducted large-scale surveillance of ticks and wild animals in the western part of the main island of Japan. We collected 6,407 host-seeking ticks from two regions and 1,598 larvae obtained from 32 engorged female ticks and examined them to elucidate transovarial transmission. In addition, we examined whole blood samples from 190 wild boars and 276 sika deer, as well as sera from 120 wild raccoons. We detected *Borrelia* spp. in *Haemaphysalis flava*, *Haemaphysalis megaspinosa*, *Haemaphysalis kitaokai*, *Haemaphysalis longicornis*, and *Haemaphysalis formosensis*. In addition, we isolated a strain from *H*. *megaspinosa* using Barbour-Stoenner-Kelly medium. The minimum infection rate of ticks was less than 5%. Transovarial transmission was observed in *H*. *kitaokai*. Phylogenetic analysis of the isolated strain and DNA fragments amplified from ticks identified at least four bacterial genotypes, which corresponded to the tick species detected. Bacteria were detected in 8.4%, 15%, and 0.8% of wild boars, sika deer, and raccoons, respectively. In this study, we found seasonal differences in the prevalence of bacterial genotypes in sika deer during the winter and summer. The tick activity season corresponds to the season with a high prevalence of animals. The present study suggests that a particular bacterial genotype detected in this study are defined by a particular tick species in which they are present.

## Introduction

Members of the genus *Borrelia* in the family Spirochaetaceae are arthropod-borne infectious agents in vertebrates [1], and they are classified into three major groups based on phylogenetic analyses: Lyme disease borreliae, relapsing fever borreliae, and reptile-associated borreliae [2, 3]. Relapsing fever borreliae are mostly found in ticks, and only *Borrelia recurrentis* is found in lice. Tick-borne relapsing fever caused by *Borrelia crocidurae*, *Borrelia duttonii*, *Borrelia hermsii*, and other related *Borrelia* spp. is a disease with worldwide distribution [4]. Tick-borne relapsing fever is mostly transmitted by soft-bodied ticks belonging to the genera *Ornithodoros* and *Argas*. By contrast, several species are transmitted by hard-bodied ticks; *Borrelia miyamotoi*, *B*. *theileri*, *Borrelia lonestari*, *Borrelia* sp. AGRF, and *Borrelia* sp. BR were detected in *Ixodes* spp., *Rhipicephalus* spp., *Amblyomma americanum*, *Amblyomma geoemydae*, and *Rhipicephalus microplus*, respectively [5–13]. In addition, a *Borrelia* sp. similar to *B*. *lonestari* was recently found in sika deer (*Cervus nippon yesoensis*) and *Haemaphysalis* spp. in Hokkaido, Japan [14, 15]. Among the hard-bodied tick-borne relapsing fever (hTBRF) borreliae, *B*. *miyamotoi* has been recognized as a human pathogen in Russia [16], the USA [17], Europe [18, 19], and Japan [20], and *B*. *theileri* has been found as the causative agent of bovine spirochetosis [6]. In the USA, *B*. *lonestari* was hypothesized to be the causative agent of southern tick-associated rash illness (STARI), which is a Lyme-like disease [21]. However, a later study did not detect *B*. *lonestari* in STARI patients [22]. Thus, the pathogenicity of *B*. *lonestari* remains unclear. Moreover, the isolation of hTBRF borreliae is difficult *in vitro*, except for *B*. *miyamotoi* from Japan and the USA and a strain of *B*. *lonestari* co-cultivated with a tick cell line [7, 23, 24]. Therefore, analyses of the genetic relationships and pathological mechanisms of hTBRF borreliae are limited.

Previously, *Borrelia* sp. detected in sika deer and *Haemaphysalis* spp. were surveyed only in Hokkaido, the northern island in Japan [14, 15]. By contrast, in the present study, we conducted large-scale surveillance of *Borrelia* spp. from ticks and wild animals in the western part of the main island of Japan. In addition, tick-derived isolates obtained from this study were subjected to molecular analyses to characterize their genetic profiles.

## Materials and methods

### Sample collection

**Fig 1:**
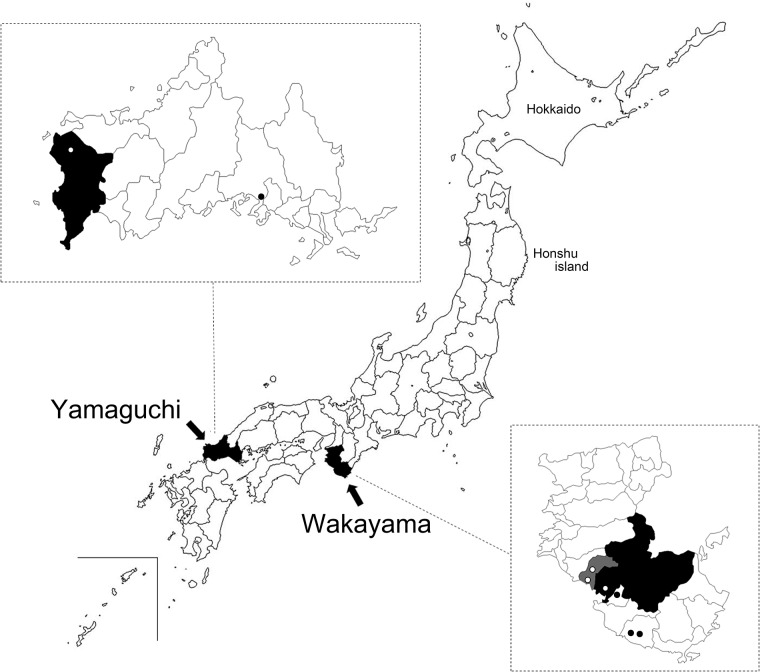
Collection sites. The gray and black shading in the lower right large-scale map indicates Minabe town and Tanabe City in Wakayama Prefecture, respectively. The black shading in the upper left large-scale map indicates Shimonoseki City in Yamaguchi Prefecture. The geographic locations of the tick sampling site are designated by black or white dots. Reprinted from (http://www.freemap.jp/item/japan/japan1.html) under a CC BY license, with permission from Keisuke Inoue, original copyright 2016.

Ticks were collected from vegetation by flagging in Wakayama and Yamaguchi prefectures from March 2014 to August 2015 (Fig 1 and S1 Table). In these areas, no specific permission was required for collecting ticks, and this study did not involve endangered or protected species. The collected ticks were identified to the species level and stage based on their morphological features [25]. To demonstrate transovarial transmission, unfed larvae were harvested from engorged female ticks collected from wild boar (*Sus scrofa*) and sika deer (*C*. *nippon*) in Shimonoseki, Yamaguchi Prefecture; the wild boars and sika deer were hunter-harvested or culled for nuisance control under the Program of Prevention from the Bird and Animal Damages from November 2013 to February 2016 (Fig 1, license number: Shimonoseki-No.24 and 26, http://www.city.shimonoseki.lg.jp/www/contents/1333690291142/files/higaiboushi.pdf). Simultaneously, whole blood and serum, as well as demographic/morphometric data including sex and estimated weight, were collected from the wild boars and sika deer. Blood samples were directly collected from the heart using a sterile needle and were dispensed into a sodium EDTA tube for DNA extraction and bacterial culture. Wild raccoons (*Procyon lotor*) were captured and culled for invasive pest control in Wakayama Prefecture during 2015 under the Program of Prevention from the Bird and Animal damages in Tanabe City and Minabe town (Fig 1, http://www.city.tanabe.lg.jp/nougyou/files/tyoujuuhigaibousikeikaku.pdf and http://www.town.minabe.lg.jp/docs/2013091300186/files/chojuboshikeikaku.pdf, respectively). Blood was directly collected from the heart during euthanasia by cardiac exsanguination under carbon dioxide anesthesia. No license was required to capture wild raccoons in Japan. No animals were killed specifically for this study. Whole blood samples and sera were stored at −20°C until further use.

### DNA extraction and cultivation from ticks

In total, 331 adult and 56 nymphal ticks from Shimonoseki, Yamaguchi Prefecture were longitudinally cut in half individually using a disposable knife, where one half was prepared for DNA extraction and the other half was used for borrelial cultivation (Table 1). DNA was extracted from one half of each tick using sodium hydroxide (NaOH) [26]. Briefly, ticks were lysed in 25 μl of 25 mM NaOH for 10 min at 95°C. Subsequently, 2 μL of Tris-HCL (1 M, pH 8.0) was added for neutralization. Cultivation was performed using modified Barbour-Stoenner-Kelly medium (BSK-M) or modified Kelly-Pettenkofer medium with 10% fetal calf serum (MKP-F) and these medium were incubated at 30°C [24, 26]. The fetal bovine serum included in this medium was replaced with inactivated fetus serum collected from sika deer at the same sampling site.

**Table 1:** Host-seeking ticks collected in Shimonoseki, Yamaguchi Prefecture.

Species	Stage	Number of samples	Number of positive samples	Prevalence
*Haemaphysalis flava*	Male	49	0	0%
	Female	29	0	0%
	Nymph	2	0	0%
*Haemaphysalis kitaokai*	Male	29	0	0%
	Female	42	0	0%
*Haemaphysalis longicornis*	Male	52	0	0%
	Female	75	0	0%
	Nymph	13	0	0%
*Haemaphysalis megaspinosa*	Male	26	1	3.85%
	Female	29	1	3.45%
	Nymph	41	0	0%
Total		387	2	0.52%

In total, 1,678 ticks from Shunan, Yamaguchi Prefecture and 4,342 ticks from Wakayama Prefecture were processed in pools of 1–50 ticks (mode: nymphs = 20, larvae = 50), thereby obtaining 685 tick pools (155 pools from Shunan and 530 pools from Wakayama) (Tables 2 and 3). The ticks were fractured using a multi-bead shocker (Yasui Kikai, Osaka, Japan) and were centrifuged at 2,500 rpm for 30 s; this procedure was repeated three times. Pellets were used for DNA extraction with a DNeasy Blood and Tissue Kit (Qiagen, Hilden, Germany) according to the manufacturer’s instructions. Eggs were harvested from 32 engorged female ticks collected from wild boar or sika deer in Shimonoseki and reared to larvae. DNA was extracted from 1, 598 larvae, processed in 32 pools of 48 to 50 (mode = 50) larvae each, by crushing them in 50 μl of 25 mM NaOH using homogenization pestles (Funakoshi Co. Ltd, Tokyo, Japan). Cultivation of the spirochete was attempted from 5 to 10 remaining larvae from an engorged female tick that produced a positive pool. DNA extracts from 1-ml whole blood samples collected from 190 wild boars and 276 sika deer were examined using a Wizard Genomic DNA Purification Kit (Promega, Madison, WI) according to the manufacturer’s instructions. DNA was extracted from the individual sera of 120 raccoons. In total, 500 μl of serum was centrifuged at 21,000 ×*g* and 4°C for 5 min, before the pellet obtained was used for DNA extraction with a DNeasy Blood and Tissue Kit (Qiagen) according to the manufacturer’s instructions. All extracted DNA samples were stored at −30°C until further use. Approximately, 100 μl of whole blood from 71 wild boars and 129 sika deer that were randomly selected was used for borrelial cultivation in 1.5 ml of BSK-M at 30°C. Cultivation was examined under dark-field microscopy (200×).

**Table 2:** Host-seeking ticks collected in Wakayama Prefecture.

Species	Stage	Number of samples	Number of pools	Number of positive pools	Minimum prevalence
*Amblyomma testudinarium*	Male	3	3	0	0%
	Female	1	1	0	0%
	Nymph	223	17	0	0%
*Dermacentor taiwanensis*	Male	2	2	0	0%
	Female	2	2	0	0%
*Ixodes ovatus*	Male	2	1	0	0%
	Female	3	2	0	0%
*Ixodes turdus*	Female	3	2	0	0%
	Nymph	8	1	0	0%
*Haemaphysalis cornigera*	Male	14	9	0	0%
	Female	10	8	0	0%
	Nymph	33	3	0	0%
*Haemaphysalis flava*	Male	96	32	4	4.17%
	Female	126	39	1	0.79%
	Nymph	490	34	4	0.82%
*Haemaphysalis formosensis*	Male	185	47	0	0%
	Female	174	48	0	0%
	Nymph	339	26	1	0.29%
*Haemaphysalis hystricis*	Male	1	1	0	0%
	Female	2	2	0	0%
*Haemaphysalis kitaokai*	Male	31	12	0	0%
	Female	38	15	1	2.63%
*Haemaphysalis longicornis*	Male	145	41	1	0.69%
	Female	136	41	0	0%
	Nymph	1,828	100	3	0.16%
*Haemaphysalis megaspinosa*	Male	16	8	0	0%
	Female	23	8	0	0%
	Nymph	408	25	6	1.47%
Total		4,342	530	21	0.48%

**Table 3:** Larval ticks prepared from engorged females collected from wild boar or sika deer in Shimonoseki, Yamaguchi Prefecture.

Species	Number of samples	Number of pools	Number of positive pools
*Amblyomma testudinarium*	50	1	0
*Haemaphysalis flava*	750	15	0
*Haemaphysalis formosensis*	50	1	0
*Haemaphysalis hystricis*	150	3	0
*Haemaphysalis kitaokai*	100	2	1
*Haemaphysalis longicornis*	50	1	0
*Haemaphysalis megaspinosa*	348	7	0
*Haemaphysalis yeni*	100	2	0
Total	1,598	32	1

### Real-time PCR of samples from ticks and wild animals

*Borrelia* spp. DNA in *Haemaphysalis* spp. were detected by real-time or quantitative PCR (qPCR) targeted at the 16S rRNA gene (*16S rDNA*) [15, 27]. Briefly, qPCR was performed with a StepOne Real-Time PCR system (Thermo Fisher Scientific, Inc., Massachusetts, US) using a Premix Ex Taq PCR kit (Probe qPCR) (TaKaRa, Shiga, Japan). The forward and reverse primers were 16S RT-F and 16S RT-R, respectively [27]. TaqMan dye-labeled minor groove binder probes BS-16S (Thermo Fisher Scientific, Inc.) was used for detecting *Borrelia* spp. in *Haemaphysalis* spp. The sensitivity of qPCR was a minimum of 10 plasmid copies [15]. qPCR was performed in a final volume of 12.5 μl for tick and 25 μl for wild animal samples. The amplification conditions were as follows: 95°C for 20 s followed by 45 cycles at 95°C for 1 s and 60°C for 20 s. The threshold line was fixed at 0.2 to avoid detecting nonspecific fluorescence.

### Conventional PCR

qPCR-positive tick samples were subjected to conventional PCR using KOD FX Neo (TOYOBO Co., Osaka, Japan). Part of the borrelial flagellin gene (*flaB*), the glycerophosphoryl diester phosphodiesterase gene *(glpQ*), and *16S rDNA* were amplified using the primer pairs BflaPAD and BflaPDU, glpQ F and glpQ R, and rrs-F1 3–26 and rrs-R4 1542–1520, respectively [2, 28]. PCR products carryover was carefully checked using distilled water as the blank control in each experiment.

For the wild boar and sika deer samples, all DNA samples were subjected to nested PCR targeted at *flaB* using Illustra PuReTaq^™^ Ready-To-Go PCR beads (GE Healthcare UK Ltd, Buckinghamshire, UK) to confirm the qPCR results. The primer sets used were BflaPAD and BflaPDU for the first PCR and BflaPBU and BflaPCR for nested PCR, as previously described [2]. qPCR-positive raccoons samples were also confirmed by *flaB* nested PCR.

All PCR products were purified using a High Pure PCR Product Purification Kit (Roche Diagnostic, Basel, Switzerland) according to the manufacturer’s instructions and were then directly sequenced with a BigDye Terminator v3.1 Cycle Sequencing Kit and an ABI3031 Genetic Analyzer (Thermo Fisher Scientific, Inc.). The primers used for detection and sequencing were listed in S2 Table. Samples that were positive according to qPCR and *flaB* sequencing were considered positive.

### Multilocus Sequence Analysis (MLSA)

MLSA was performed with isolates using the primer sets listed in S2 Table based on the loci from eight genes (*clpA*, *clpX*, *nifS*, *pepX*, *pyrG*, *recG*, *rplB*, and *uvrA*), as previously described [26]. PCR was conducted at 94°C for 30 s followed by 45 cycles at 94°C for 30 s, 50°C for 30 s, and 72°C for 30 s. Sequencing was performed as described above.

### Phylogenetic analysis

The sequences of *flaB*, *glpQ*, and *16S rDNA* were aligned by CLUSTALW, and neighbor-joining trees were generated based on 1,000 bootstrap replicates according to the Kimura two-parameter methods in MEGA 5.2 (http://www.megasoftware.net) [29]. All positions containing alignment gaps and missing nucleotides were eliminated only in pairwise sequence comparisons (the pairwise deletion option was used). After concatenating the sequences, the MLSA phylogenetic tree was constructed based on Bayesian phylogenetic analysis, as previously described [30, 31]. CLUSTALX was used to align the sequences, and phylogenetic analysis was performed with MrBayes 3.2.2 [30, 31]. The general time-reversible model with site-specific rates was used as the evolutionary model for analyses in MrBayes. The first, second, and third codon positions were defined for coding sequences. Analyses were continued for 5 × 10^7^ generations or until the average standard deviation of the split frequencies was <0.01. Data were sampled every 100th generation for genes subjected to MLSA. A phylogenetic tree was constructed for MLSA sequences using FigTree v1.4.2. The genetic mean pairwise distance between bacterial genotypes was calculated using the Kimura two-parameter model in MEGA5.2. All sequence data have been deposited in DDBJ/EMBL/GenBank (accession numbers LC170019 to LC170035 and LC171370 to LC171377), and reference sequences were downloaded from DDBJ/EMBL/GenBank or the MLST database (http://www.mlst.net/databases/).

### Statistical analysis

Significant differences in prevalence were determined using Fisher’s exact test. *P*-values of <0.05 were considered significant.

## Results

We collected 6,407 host-seeking ticks from Yamaguchi and Wakayama prefectures (Fig 1 and S1 Table). From Shimonoseki, Yamaguchi Prefecture, we collected 387 *Haemaphysalis* ticks, which were individually prepared for DNA detection and cultivation (Table 1). Borrelial DNA fragments were detected in two *H*. *megaspinosa* ticks (a male and female), and the prevalence was 3.6% (2/55) in adult *H*. *megaspinosa*. In these PCR-positive ticks, a strain was successfully isolated from female ticks using BSK-M, and the strain was designated as tHM16w. This is the first *Borrelia* sp. isolate detected in *Haemaphysalis* ticks using BSK-M. In Shunan, Yamaguchi Prefecture, 1,678 ticks in 155 pools were examined and borrelial DNA was not detected. In Wakayama, 4,342 ticks were collected and processed in 530 pools (Table 2). Among the 530 tick pools, 21 were positive and the minimum prevalence was 0.48% (21/4,342): four, one, and four pools from *H*. *flava* males, females, and nymphs (4/96, 1/126, and 4/490, and minimum prevalence of 4.17%, 0.79%, and 0.82%), respectively; one pool from *H*. *formosensis* nymphs (1/339; 0.29%); one pool from *H*. *kitaokai* females (1/38; 2.63%); one and three pools from *H*. *longicornis* males and nymphs (1/145 and 3/1,828; 0.69% and 0.16%), respectively; and six pools from *H*. *megaspinosa* nymph (6/408; 1.47%). We also examined unfed larval ticks prepared from engorged females. From 32 engorged females, we examined 1,598 larval ticks in 32 pools (Table 3). *Borrelia* was not isolated using BSK-M, but a DNA fragment was detected in a pool from *H*. *kitaokai*.

**Fig 2:**
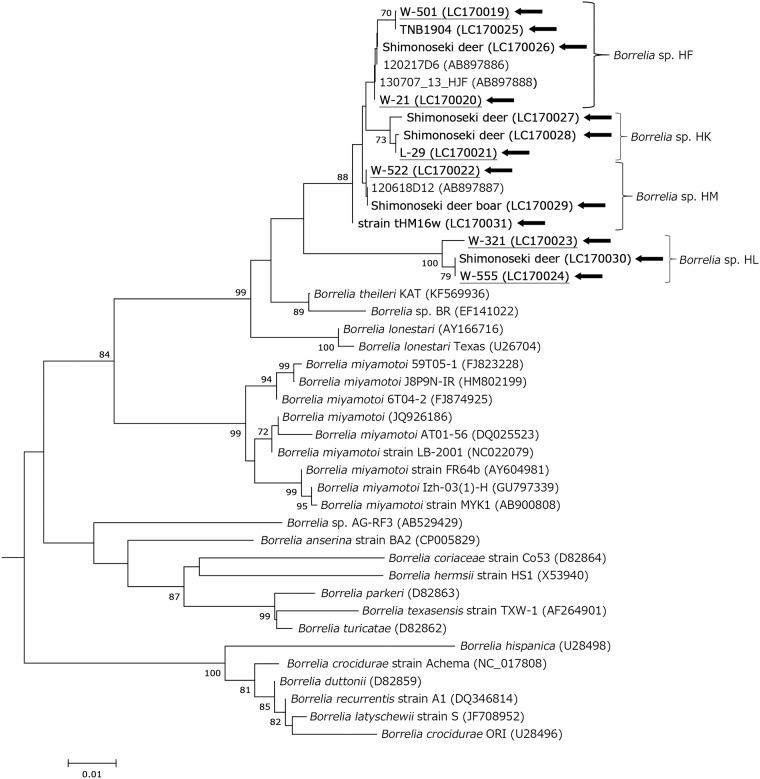
Phylogenetic analysis of *flaB* in *Borrelia* spp. The tree was constructed using the neighbor-joining method based on the Kimura two-parameter model. The phylogenetic branches were supported by >70% according to the bootstrap analysis. The bar indicates the percentage of sequence divergence. *B*. *afzelii* VS461 (accession no. D63365), *B*. *burgdorferi* B31 (AB035617), and *B*. *garinii* 20047 (AB035602) were used as outgroups (data not indicated). Pointing arrows and bold type indicate the results obtained in the present study. TNB1904 is the raccoon sample from Wakayama Prefecture. Underlined samples were derived from ticks. Numbers in parentheses represent GenBank accession numbers.

**Fig 3:**
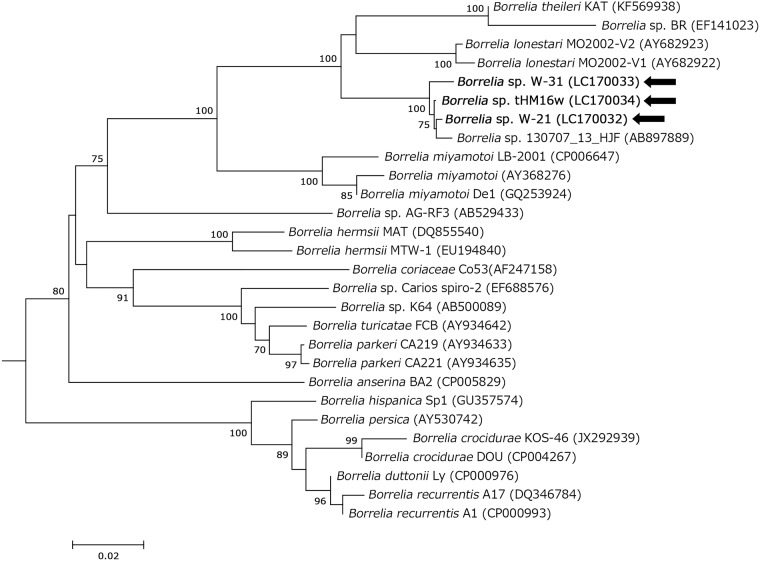
Phylogenetic analysis of *glpQ* in *Borrelia* spp. The tree was constructed using the neighbor-joining method based on the Kimura two-parameter model. The phylogenetic branches were supported by >70% according to the bootstrap analysis. The bar indicates the percentage of sequence divergence. *Borrelia* sp. BF-16 (accession no. AB529436), *Borrelia* sp. Tick98M (AB529432), *Borrelia* sp. TA2 (AB529434), and *Borrelia* sp. Tortoise14H1 (AB529431) were used as outgroups (data not indicated). Pointing arrows and bold type indicate the results obtained in the present study. Numbers in parentheses represent GenBank accession numbers.

**Fig 4:**
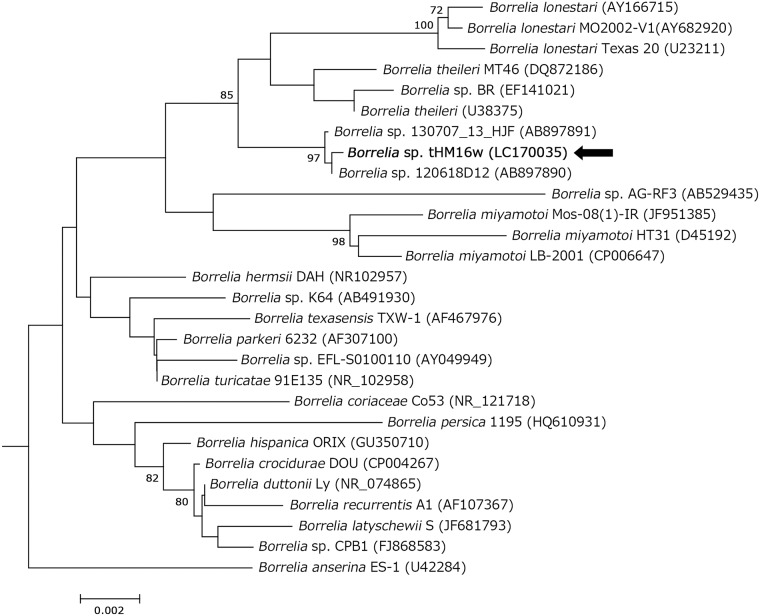
Phylogenetic analysis of *16S rDNA* in *Borrelia* spp. The tree was constructed using the neighbor-joining method based on the Kimura two-parameter model. The phylogenetic branches were supported by >70% according to the bootstrap analysis. The bar indicates the percentage of sequence divergence. *B*. *afzelii* PKo (accession no. NR_074840), *B*. *garinii* PBi (CP000013), and *B*. *burgdorferi* B31 (U03396) were used as outgroups (data not indicated). Pointing arrows and bold type indicate the results obtained in the present study. Numbers in parentheses represent GenBank accession numbers.

Based on the phylogenetic analysis of *flaB* sequences obtained from ticks, the *Borrelia* spp. detected formed a different branch compared with *B*. *theileri* and *B*. *lonestari* (Fig 2). Moreover, the borreliae we detected were phylogenetically distinguished according to the tick species in which they were detected. Therefore, we preliminarily designated the bacterial genotypes detected in *H*. *flava*, *H*. *kitaokai*, *H*. *longicornis*, and *H*. *megaspinosa* as *Borrelia* sp. HF, *Borrelia* sp. HK, *Borrelia* sp. HL, and *Borrelia* sp. HM, respectively (Fig 2). The bacterial genotypes and tick species detected are summarized in S3 Table. All bacterial genotypes were detected in each representative tick species, except *for Borrelia* sp. HF that was detected in two nymphal pools from *H*. *formosensis* and *H*. *longicornis* (S3 Table). The group mean pairwise distances for *flaB* among the four bacterial genotypes are shown in S4 Table. Sequencing and phylogenetic analyses of the housekeeping gene *glpQ* (1011 bp) were performed using two *Borrelia* sp. HK samples (W-31 and L-29, identical), one *Borrelia* sp. HF sample (W-21), and the *Borrelia* sp. HM tHM16w isolate (Fig 3), as well as of *16S rDNA* (1490 bp) using one *Borrelia* sp. HM tHM16w isolate (Fig 4). The *16S rDNA* sequence of *Borrelia* sp. HM tHM16w was identical to that of the *Borrelia* sp. detected in sika deer in Hokkaido (AB897890) but was slightly different from that of AB897891, which was detected in *Haemaphysalis japonica*, where there was one nucleotide substitution. The group mean pairwise distances for *glpQ* and *16S rDNA* compared with *B*. *theileri* and *B*. *lonestari* are shown in S5 Table. According to the group mean pairwise distances and phylogenetic analyses of housekeeping genes, the *Borrelia* spp. detected were genetically more similar to *B*. *theileri* than to *B*. *lonestari* (Figs 2–4 and S5 Table). A phylogenetic tree was constructed by Bayesian phylogenetic inference using the MLSA sequences of isolate tHM16w (S1 Fig), which showed that the isolate clustered with other hTBRF borreliae such as *B*. *miyamotoi*.

Among the 190 wild boars and 276 sika deer captured in Shimonoseki, 16 and 42 individuals, respectively, were positive for *Borrelia* spp. according to qPCR and sequencing analysis of *flaB* (infection rate = 8.4% and 15%, respectively) (Tables 4 and 5). All positive samples from wild boars were infected with *Borrelia* sp. HM. However, the sika deer were infected with four bacterial genotypes: *Borrelia* sp. HF was detected in 5 samples (5/276, infection rate = 1.8%), *Borrelia* sp. HK in 11 (11/276, 4%), *Borrelia* sp. HM in 12 (12/276, 4.3%), and *Borrelia* sp. HL in 11 samples (11/276, 4%). The remaining three samples from sika deer may have been co-infected with two or more bacterial genotypes according to the sequencing analysis. No borrelial cells were isolated from whole blood samples from wild boars and sika deer. There were no significant differences in the prevalence of *Borrelia* sp. HM and *Borrelia* sp. HF in the summer (from April to September) or winter (from October to March). However, there were seasonal differences during the winter and summer in the prevalence of *Borrelia* sp. HK (winter; 7.2%, summer; 0.7%, *P* = 0.0102) and *Borrelia* sp. HL (winter; 0%, summer; 8%, *P* = 0.0008). We also examined DNA extracted from the sera of wild raccoons in Wakayama using qPCR. Among the 120 samples, one sample was positive for *Borrelia* sp. HF (1/120, infection rate = 0.83%) (S6 Table). The sequences detected in wild animals collected in Yamaguchi or Wakayama Prefecture clustered in the same branch for each bacterial genotype (Fig 2).

**Table 4:** Prevalence of *Borrelia* sp. in wild boars collected from Shimonoseki, Yamaguchi Prefecture.

Month	Number of samples	Number of positive samples (prevalence %)					
		*Borrelia* sp. HF	*Borrelia* sp. HK	*Borrelia* sp. HM	*Borrelia* sp. HL	Co-infection	Total
Jan	27	0	0	1	0	0	1 (3.7%)
Feb	14	0	0	1	0	0	1 (7.1%)
Mar	11	0	0	0	0	0	0
Apr	19	0	0	2	0	0	2 (11%)
May	10	0	0	1	0	0	1 (10%)
Jun	6	0	0	0	0	0	0
Jul	6	0	0	2	0	0	2 (33%)
Aug	13	0	0	0	0	0	0
Sep	12	0	0	0	0	0	0
Oct	14	0	0	1	0	0	1 (7.1%)
Nov	22	0	0	2	0	0	2 (9.1%)
Dec	36	0	0	6	0	0	6 (17%)
Total	190	0	0	16 (8.4%)	0	0	16 (8.4%)

**Table 5:** Prevalence of *Borrelia* sp. in sika deer collected from Shimonoseki, Yamaguchi Prefecture.

Month	Number of samples	Number of positive samples (prevalence %)					
		*Borrelia* sp. HF	*Borrelia* sp. HK	*Borrelia* sp. HM	*Borrelia* sp. HL	Co-infection	Total
Jan	14	1	2	0	0	0	3 (21%)
Feb	34	0	6	3	0	1	10 (29%)
Mar	49	0	0	4	0	1	5 (10%)
Apr	26	0	1	2	3	0	6 (23%)
May	26	0	0	1	2	0	3 (12%)
Jun	28	0	0	0	3	0	3 (11%)
Jul	21	0	0	0	2	0	2 (9.5%)
Aug	21	0	0	0	1	1	2 (9.5%)
Sep	16	0	0	0	0	0	0
Oct	15	1	0	2	0	0	3 (20%)
Nov	13	2	0	0	0	0	2 (15%)
Dec	13	1	2	0	0	0	3 (23%)
Total	276	5 (1.8%)	11 (4.0%)	12 (4.3%)	11 (4.0%)	3 (1.1%)	42 (15%)

## Discussion

We detected several bacterial genotypes of *Borrelia* spp. from *Haemaphysalis* spp. collected from two regions in the western part of the main island of Japan. The prevalence was 0%–0.5% in all ticks collected and 0%–4% in each tick species (Tables 1 and 2). In a previous study, Lee et al. found that the prevalence of *Borrelia* spp. in *Haemaphysalis* adult ticks was 0.7% [15]. Several surveys of *B*. *lonestari* derived from unfed *A*. *americanum* adults have shown that the prevalence in the USA was less than 6% [32–37]. However, *B*. *theileri* has been detected in host-attached ticks. McCoy et al. reported that the prevalence of *B*. *theileri* in cattle infested with *Rhipicephalus geigyi* was 0.5% in Mari, while Cutler et al. detected *B*. *theileri* in 12.5% pools of animal-associated *Amblyomma* and *Rhipicephalus* spp. in Ethiopia [38, 39]. Moreover, the prevalence of human pathogenic hTBRF borreliae, *B*. *miyamotoi*, in unfed ixodid ticks was less than 5% in the USA, Eurasia, and Japan [26, 40, 41]. Our results and those of previous investigations suggest that the prevalence of hTBRF borreliae in unfed ticks is generally less than 5%. Transovarial transmission of *Borrelia* sp. HK in *H*. *kitaokai* was demonstrated in the present study (Table 3), which has also been examined in *B*. *lonestari* and *B*. *miyamotoi* [35, 42]. Thus, we suggest that hTBRF borreliae might be maintained in the environment via transovarial transmission.

We elucidated four bacterial genotypes among the *Borrelia* spp. derived from *Haemaphysalis* ticks (Fig 2 and S3 Table). The ticks were collected from different regions, but each bacterial genotype was detected mostly in a specific tick species. Moreover, *Borrelia* sp. HM detected in ticks and wild animals in Shimonoseki in the western part of Japan was not genetically distinguished from that detected in Hokkaido in the northern island of Japan in a previous study (Figs 1 and 2, Accession Number AB897887) [15]. In most cases, the genus *Borrelia* was transmitted by a particular tick species [4]. Thus, our results suggest that the bacterial genotypes detected in this study might be defined by the tick species from which they were detected. The group mean pairwise distances between *Borrelia* sp. HF, *Borrelia* sp. HK, and *Borrelia* sp. HM showed that they slightly differed in terms of *flaB* (mean pairwise distances over 99%). We analyzed other housekeeping genes (Figs 3 and 4), but some genes could not be amplified using the representative primer sets. Recently, MLSA was used for the intra- or inter-species characterization of Lyme disease borreliae and some hTBRF borreliae [26, 30, 31, 43]. We used MLSA to analyze *Borrelia* sp. HM strain tHM16w (S1 Fig), but the amplification efficiency was low, even when we used strain-derived DNA. Moreover, *nifS*, *pepX* and *uvrA* were not amplified when we used DNA prepared from ticks (data not shown). These results may be due to mismatches in the primers, which were originally designed based on representative borrelial species. Further analysis such as genome sequencing will be required to define the genetic characteristics of the bacterial genotypes detected in this study.

In a previous study, Lee et al. showed that 10.6% of sika deer (*C*. *nippon yesoensis*) were infected with *Borrelia* sp. in Hokkaido [15]. In the USA, a DNA fragment from *B*. *lonestari* was detected in 8.7% or 3.1% of blood samples from white-tailed deer (*Odocoileus virginianus*), 13% of samples from Eastern wild turkey (*Meleagris gallopavo silvestris*), and 7.4% of samples from migratory waterfowl [American black buck (*Anas rubripes*), Canada goose (*Branta canadensis*), mallard (*Anas platyrhynchos*), northern pintail (*Anas acuta*), ring-necked duck (*Aythya collaris*), and wood duck (*Aix sponsa*)] [44–46]. However, no borrelial DNA was detected in raccoons from the USA, although antibodies were detected [47]. In our survey, borrelial DNA was detected in 8.4% of wild boars, 15% of sika deer, and 0.83% of wild raccoons (Tables 4 and 5 and S6 Table). In this present study, we detected borrelial DNA using a serum sample in case of raccoons. The difference in collection may affect the low prevalence in raccoons. On the other hand, most relapsing fever borreliae were usually detected in whole blood and serum because of high bacteremia [48]. Moreover, *B*. *miyamotoi* DNA was detected in human serum [20]. From our result and previous observations, we speculated that the *Borrelia* spp. found in this study and *B*. *theileri* infect the order Artiodactyla. We did not detect *Borrelia* spp. in ticks collected from a park in Shunan, where wild boars and sika deer were absent and birds were present. On the other hand, we could not isolate the bacteria in blood samples. Lee et al. reported the average of bacteremia in sika deer blood to be 3.5 in log_10_ per ml [15]. Because we observed 0.1ml blood for cultivation, the low copy number of bacteria in blood might be involved in this result.

In Shimonoseki, where we collected sika deer, it was reported that sika deer were infested with *H*. *longicornis*, *Haemaphysalis yeni*, *H*. *flava*, *H*. *megaspinosa*, *H*. *kitaokai*, *Ixodes ovatus*, and *Amblyomma testudinarium*. In addition, it was reported that *H*. *flava* and *H*. *megaspinosa* are active in all seasons. *H*. *kitaokai* and *H*. *longicornis* exhibit seasonal changes in their activity; they are mainly active in the winter and summer, respectively [49]. In this study, we found seasonal differences in the prevalence of bacterial genotypes in sika deer during the winter and summer, i.e., *Borrelia* sp. HK (detected from *H*. *kitaokai*) was detected in the winter and *Borrelia* sp. HL (detected from *H*. *longicornis*) was detected in the summer. The tick activity season corresponds to the season with a high prevalence of animals; therefore, we suggest that *Borrelia* sp. HK and *Borrelia* sp. HL were transiently infected and that there was no chronic bacteremia in sika deer. We only detected *Borrelia* sp. HM in wild boars, and *H*. *flava*, *H*. *megaspinosa* and *A*. *testudinarium* infested wild boars in our sampling site (data not shown). Moreover, *H*. *flava*, *H*. *longicornis*, and *A*. *testudinarium* infested wild boars in Shimane Prefecture, which neighbors Yamaguchi Prefecture [50]. Therefore, we suggest that wild boars are infected with a limited range of bacterial genotypes.

In this study, we detected *Borrelia* sp. derived from *Haemaphysalis* ticks in two regions in the western part of the main island of Japan. We detected four bacterial genotypes in *Haemaphysalis* ticks and wild animals. Our results suggest that the bacterial genotypes detected in this study are defined by the tick species in which they are present.

### Originality-significance statement

The authors confirm that all of this reported work is original. This is the first report to show the prevalence of *Haemaphysalis* ticks associated borreliae in ticks and wild animals in the western part of the main island of Japan.

## Supporting information

S1 Table The sampling site of ticks in this study. (DOCX)

S2 Table Primer list. (DOCX)

S3 Table The bacterial genotype and tick species positive for *Borrelia* spp. in this study. (DOCX)

S4 Table Genetic group mean pairwise distance for *flaB* among 4 types of *Borrelia* spp. in this study. (DOCX)

S5 Table Genetic group mean distance of *16S rDNA* (right upper) or *glpQ* (left lower) of *Borrelia* spp. in this study and other hard-bodied tick-borne relapsing fever borreliae. (DOCX)

S6 Table Prevalence of *Borrelia* sp. in wild raccoons collected from Wakayama Prefecture. (DOCX)

S1 Fig Bayesian phylogenetic analysis of borrelial housekeeping gene sequences. (DOCX)

## References

[1] Parola P, Raoult D (2001) Ticks and tickborne bacterial diseases in humans: an emerging infectious threat. Clin Infect Dis 32(6): 897–928. 10.1086/319347 11247714

[2] Takano A, Goka K, Une Y, Shimada Y, Fujita H, Shiino T, et al (2010) Isolation and characterization of a novel *Borrelia* group of tick-borne borreliae from imported reptiles and their associated ticks. Environ Microbiol 12(1): 134–46. 10.1111/j.1462-2920.2009.02054.x 19758349

[3] Franke J, Hildebrandt A, Dorn W (2013) Exploring gaps in our knowledge on Lyme borreliosis spirochaetes—updates on complex heterogeneity, ecology, and pathogenicity. Ticks Tick Borne Dis 4(1–2): 11–25. 10.1016/j.ttbdis.2012.06.007 23246041

[4] Barbour AG (2005) Relapsing fever In: Goodman JL, Dennis DT, Sonenshine DE, editors. Tick-borne diseases of humans: ASM Press pp. 268–91.

[5] Trees AJ (1978) The transmission of *Borrelia theileri* by *Boophilus annulatus* (Say, 1821). Trop Anim Health Prod 10(2): 93–4. 664021 10.1007/BF02235315

[6] Smith RD, Miranpuri GS, Adams JH, Ahrens EH (1985) *Borrelia theileri*: isolation from ticks (*Boophilus microplus*) and tick-borne transmission between splenectomized calves. Am J Vet Res 46(6): 1396–8. 4026019

[7] Fukunaga M, Takahashi Y, Tsuruta Y, Matsushita O, Ralph D, et al (1995) Genetic and phenotypic analysis of *Borrelia miyamotoi* sp. nov., isolated from the ixodid tick *Ixodes persulcatus*, the vector for Lyme disease in Japan. Int J Syst Bacteriol 45: 804–10. 10.1099/00207713-45-4-804 7547303

[8] Barbour AG, Maupin GO, Teltow GJ, Carter CJ, Piesman J (1996) Identification of an uncultivable *Borrelia* species in the hard tick *Amblyomma americanum*: possible agent of a Lyme disease-like illness. J Infect Dis 173(2): 403–9. 8568302 10.1093/infdis/173.2.403

[9] Scoles GA, Papero M, Beati L, Fish D (2001) A relapsing fever group spirochete transmitted by *Ixodes scapularis* ticks. Vector Borne Zoonotic Dis 1: 21–34. 10.1089/153036601750137624 12653133

[10] Fraenkel CJ, Garpmo U, Berglund J (2002) Determination of novel *Borrelia* genospecies in Swedish *Ixodes ricinus* ticks. J Clin Microbiol 40: 3308–12. 10.1128/JCM.40.9.3308-3312.2002 12202571 PMC130762

[11] Mun J, Eisen RJ, Eisen L, Lane RS (2006) Detection of a *Borrelia miyamotoi* sensu lato relapsing-fever group spirochete from *Ixodes pacificus* in California. J Med Entomol 43: 120–3. 16506458 10.1093/jmedent/43.1.120

[12] Yparraguirre LA, Machado-Ferreira E, Ullmann AJ, Piesman J, Zeidner NS, Soares CA (2007) A hard tick relapsing fever group spirochete in a Brazilian *Rhipicephalus* (*Boophilus*) *microplus*. Vector Borne Zoonotic Dis 7(4): 717–21. 10.1089/vbz.2007.0144 17979536

[13] Takano A, Sugimori C, Fujita H, Kadosaka T, Taylor KR, Tsubota T, et al (2012) A novel relapsing fever *Borrelia* sp. infects the salivary glands of the molted hard tick, *Amblyomma geoemydae*. Ticks Tick Borne Dis 3(4): 259–61. 10.1016/j.ttbdis.2012.06.003 22910061

[14] Taylor, K.R. The ecologies of Borrelia spp. in Hokkaido, Japan. PhD thesis, Hokkaido University, Hokkaido, Japan. 2013. http://hdl.handle.net/2115/52271

[15] Lee K, Takano A, Taylor K, Sashika M, Shimozuru M, Konnai S, et al (2014) A relapsing fever group *Borrelia* sp. similar to *Borrelia lonestari* found among wild sika deer (*Cervus nippon yesoensis*) and *Haemaphysalis* spp. ticks in Hokkaido, Japan. Ticks Tick Borne Dis 5(6): 841–7. 10.1016/j.ttbdis.2014.06.006 25108784

[16] Platonov AE, Karan LS, Kolyasnikova NM, Makhneva NA, Toporkova MG, Maleev VV, et al (2011) Humans infected with relapsing fever spirochete *Borrelia miyamotoi*, Russia. Emerg Infect Dis 17(10): 1816–23. 10.3201/eid1710.101474 22000350 PMC3310649

[17] Chowdri HR, Gugliotta JL, Berardi VP, Goethert HK, Molloy PJ, Sterling SL, et al (2013) *Borrelia miyamotoi* infection presenting as human granulocytic anaplasmosis: a case report. Ann Intern Med 159(1): 21–7. 10.7326/0003-4819-159-1-201307020-00005 23817701

[18] Hovius JW, de Wever B, Sohne M, Brouwer MC, Coumou J, Wagemakers A, et al (2013) A case of meningoencephalitis by the relapsing fever spirochaete *Borrelia miyamotoi* in Europe. Lancet 382(9892): 658 10.1016/S0140-6736(13)61644-X 23953389 PMC3987849

[19] Boden K, Lobenstein S, Hermann B, Margos G, Fingerle V (2016) *Borrelia miyamotoi*-Associated Neuroborreliosis in Immunocompromised Person. Emerg Infect Dis 22(9): 1617–20. 10.3201/eid2209.152034 27533748 PMC4994329

[20] Sato K, Takano A, Konnai S, Nakao M, Ito T, Koyama K, et al (2014) Human infections with *Borrelia miyamotoi*, Japan. Emerg Infect Dis 20(8): 1391–3. 10.3201/eid2008.131761 25061761 PMC4111186

[21] James AM, Liveris D, Wormser GP, Schwartz I, Montecalvo MA, Johnson BJ (2001) *Borrelia lonestari* infection after a bite by an *Amblyomma americanum* tick. J Infect Dis 183(12): 1810–4. 10.1086/320721 11372036

[22] Wormser GP, Masters E, Liveris D, Nowakowski J, Nadelman RB, Holmgren D, et al (2005) Microbiologic evaluation of patients from Missouri with erythema migrans. Clin Infect Dis 40(3): 423–8. 10.1086/427289 15668867 PMC2773674

[23] Varela AS, Luttrell MP, Howerth EW, Moore VA, Davidson WR, Stallknecht DE, et al (2004) First culture isolation of *Borrelia lonestari*, putative agent of southern tick-associated rash illness. J Clin Microbiol 42(3): 1163–9. 10.1128/JCM.42.3.1163-1169.2004 15004069 PMC356874

[24] Wagemakers A, Oei A, Fikrig MM, Miellet WR, Hovius JW (2014) The relapsing fever spirochete Borrelia miyamotoi is cultivable in a modified Kelly-Pettenkofer medium, and is resistant to human complement. Parasit Vectors 7: 418 10.1186/1756-3305-7-418 25189195 PMC4261524

[25] Yamaguti N, Tipton V, Keegan H, Toshioka S (1971) Ticks of Japan, Korea, and the Ryukyu Islands, vol. 15 Brigham Young University Science Bulletin, Provo, UT pp. 1–227.

[26] Takano A, Toyomane K, Konnai S, Ohashi K, Nakao M, Ito T, et al (2014) Tick surveillance for relapsing fever spirochete *Borrelia miyamotoi* in Hokkaido, Japan. PLoS One 9(8): e104532 10.1371/journal.pone.0104532 25111141 PMC4128717

[27] Barbour AG, Bunikis J, Travinsky B, Hoen AG, Diuk-Wasser MA, et al (2009) Niche partitioning of *Borrelia burgdorferi* and *Borrelia miyamotoi* in the same tick vector and mammalian reservoir species. Am J Trop Med Hyg 81: 1120–31. 10.4269/ajtmh.2009.09-0208 19996447 PMC2841027

[28] Bacon RM, Pilgard MA, Johnson BJ, Raffel SJ, Schwan TG (2004) Glycerophosphodiester phosphodiesterase gene (*glpQ*) of *Borrelia lonestari* identified as a target for differentiating *Borrelia* species associated with hard ticks (Acari:Ixodidae). J Clin Microbiol 42(5): 2326–2328. 10.1128/JCM.42.5.2326-2328.2004 15131225 PMC404625

[29] Tamura K, Peterson D, Peterson N, Stecher G, Nei M, Kumar S (2011) MEGA5: molecular evolutionary genetics analysis using maximum likelihood, evolutionary distance, and maximum parsimony methods. Mol Biol Evol. 28(10): 2731–9. 10.1093/molbev/msr121 21546353 PMC3203626

[30] Margos G, Gatewood AG, Aanensen DM, Hanincová K, Terekhova D, Vollmer SA, et al (2008) MLST of housekeeping genes captures geographic population structure and suggests a European origin of *Borrelia burgdorferi*. Proc Natl Acad Sci U S A. 105(25): 8730–5. 10.1073/pnas.0800323105 18574151 PMC2435589

[31] Margos G, Vollmer SA, Cornet M, Garnier M, Fingerle V, Wilske B, et al (2009) A new *Borrelia* species defined by multilocus sequence analysis of housekeeping genes. Appl Environ Microbiol. 75(16): 5410–6. 10.1128/AEM.00116-09 19542332 PMC2725479

[32] Mixson TR, Campbell SR, Gill JS, Ginsberg HS, Reichard MV, Schulze TL, et al (2006) Prevalence of *Ehrlichia*, *Borrelia*, and *Rickettsial* agents in *Amblyomma americanum* (Acari: Ixodidae) collected from nine states. J Med Entomol. 43(6): 1261–8. 17162962 10.1603/0022-2585(2006)43[1261:poebar]2.0.co;2

[33] Schulze TL, Jordan RA, Healy SP, Roegner VE, Meddis M, Jahn MB, et al (2006) Relative abundance and prevalence of selected *Borrelia* infections in *Ixodes scapularis* and *Amblyomma americanum* (Acari: Ixodidae) from publicly owned lands in Monmouth County, New Jersey. J Med Entomol. 43(6): 1269–75. 17162963 10.1603/0022-2585(2006)43[1269:raapos]2.0.co;2

[34] Castellaw AH, Showers J, Goddard J, Chenney EF, Varela-Stokes AS (2010) Detection of vector-borne agents in lone star ticks, *Amblyomma americanum* (Acari: Ixodidae), from Mississippi. J Med Entomol 47(3): 473–6. 20496596 10.1603/me09263

[35] Killmaster LF, Loftis AD, Zemtsova GE, Levin ML (2014) Detection of bacterial agents in *Amblyomma americanum* (Acari: Ixodidae) from Georgia, USA, and the use of a multiplex assay to differentiate *Ehrlichia chaffeensis* and *Ehrlichia ewingii*. J Med Entomol 51(4): 868–72. 25118421 10.1603/me13225 PMC5659119

[36] Maegli A, Loy JD, Cortinas R (2016) Note on *Ehrlichia chaffeensis*, *Ehrlichia ewingii*, and "*Borrelia lonestari*" infection in lone star ticks (Acari: Ixodidae), Nebraska, USA. Ticks Tick Borne Dis 7(1): 154–8. 10.1016/j.ttbdis.2015.10.008 26515060

[37] Sayler KA, Loftis AD, Beatty SK, Boyce CL, Garrison E, Clemons B, et al (2016) Prevalence of Tick-Borne Pathogens in Host-Seeking *Amblyomma americanum* (Acari: Ixodidae) and *Odocoileus virginianus* (Artiodactyla: Cervidae) in Florida. J Med Entomol 53(4): 949–956. 10.1093/jme/tjw054 27117680

[38] Cutler S, Abdissa A, Adamu H, Tolosa T, Gashaw A (2012) *Borrelia* in Ethiopian ticks. Ticks Tick Borne Dis 3(1): 14–7. 10.1016/j.ttbdis.2011.08.004 22309854

[39] McCoy BN, Maïga O, Schwan TG (2014) Detection of *Borrelia theileri* in *Rhipicephalus geigyi* from Mali. Ticks Tick Borne Dis 5(4): 401–3. 10.1016/j.ttbdis.2014.01.007 24709337 PMC4041617

[40] Wagemakers A, Staarink PJ, Sprong H, Hovius JW (2015) *Borrelia miyamotoi*: a widespread tick-borne relapsing fever spirochete. Trends Parasitol 31(6): 260–9. 10.1016/j.pt.2015.03.008 25892254

[41] Siński E, Welc-Falęciak R, Zajkowska J (2016) *Borrelia miyamotoi*: A human tick-borne relapsing fever spirochete in Europe and its potential impact on public health. Adv Med Sci 61(2): 255–60. 10.1016/j.advms.2016.03.001 27100337

[42] Rollend L, Fish D, Childs JE (2013) Transovarial transmission of *Borrelia* spirochetes by *Ixodes scapularis*: a summary of the literature and recent observations. Ticks Tick Borne Dis 4(1–2): 46–51. 10.1016/j.ttbdis.2012.06.008 23238242

[43] Margos G, Wilske B, Sing A, Hizo-Teufel C, Cao WC, Chu C, et al (2013) *Borrelia bavariensis* sp. nov. is widely distributed in Europe and Asia. Int J Syst Evol Microbiol 63(Pt 11): 4284–8. 10.1099/ijs.0.052001-0 23838444

[44] Moore VA IV, Varela AS, Yabsley MJ, Davidson WR, Little SE (2003) Detection of *Borrelia lonestari*, putative agent of southern tick-associated rash illness, in white-tailed deer (*Odocoileus virginianus*) from the southeastern United States. J Clin Microbiol 41(1): 424–7. 10.1128/JCM.41.1.424-427.2003 12517884 PMC149587

[45] Castellaw AH, Chenney EF, Varela-Stokes AS (2011) Tick-borne disease agents in various wildlife from Mississippi. Vector Borne Zoonotic Dis 11(4): 439–42. 10.1089/vbz.2009.0221 20846016

[46] Jordan BE, Onks KR, Hamilton SW, Hayslette SE, Wright SM (2009) Detection of *Borrelia burgdorferi* and *Borrelia lonestari* in birds in Tennessee. J Med Entomol 46(1): 131–8. 19198527 10.1603/033.046.0117

[47] Yabsley MJ, Murphy SM, Luttrell MP, Little SE, Massung RF, Stallknecht DE, et al (2008) Experimental and field studies on the suitability of raccoons (*Procyon lotor*) as hosts for tick-borne pathogens. Vector Borne Zoonotic Dis 8(4): 491–503. 10.1089/vbz.2007.0240 18429696

[48] Kutsuna S, Kawabata H, Kasahara K, Takano A, Mikasa K (2013) The first case of imported relapsing fever in Japan. Am J Trop Med Hyg 89(3): 460–1. 10.4269/ajtmh.13-0187 23857020 PMC3771281

[49] Inokuma H, Fujimoto T, Hosoi E, Tanaka S, Fujisaki K, Okuda M, et al (2002) Tick infestation of sika deer (*Cervus nippon*) in the western part of Yamaguchi Prefecture, Japan. J Vet Med Sci 64(7): 615–7. 12185317 10.1292/jvms.64.615

[50] Motoi Y, Asano M, Inokuma H, Ando S, Kawabata H, Takano A, et al (2013) Detection of *Rickettsia tamurae* DNA in ticks and wild boar (*Sus scrofa leucomystax*) skins in Shimane Prefecture, Japan. J Vet Med Sci 75(3): 263–7. 23075922 10.1292/jvms.12-0299

