# Supplementary material for: Epidemiological study of relapsing fever borreliae detected in Haemaphysalis ticks and wild animals in the western part of Japan
Source: PLoS One. 2017 Mar 31;12(3):e0174727. doi: 10.1371/journal.pone.0174727 (PMC5375152; doi:10.1371/journal.pone.0174727)
Supplement: S1 Table — (DOCX) [file pone.0174727.s001.docx]

**S1 Table. The sampling site of ticks in this study.**

| Prefecture | Local name | North latitude | East longitude |
| --- | --- | --- | --- |
| Yamaguchi | Shimonoseki | 34°18′06′′ | 130°58′52′′ |
|  | Shunan | 34°02′21′′ | 131°49′56′′ |
| Wakayama | Susami 1 | 33°33′49′′ | 135°31′03′′ |
|  | Susami 2 | 33°33′08′′ | 135°35′45′′ |
|  | Toyoakitsu | 33°44′58′′ | 135°23′38′′ |
|  | Minabe 1 | 33°49′30′′ | 135°19′32′′ |
|  | Minabe 2 | 33°46′20′′ | 135°18′26′′ |
|  | Kamitonda | 33°44′13′′ | 135°27′07′′ |
