# Supplementary material for: Epidemiological study of relapsing fever borreliae detected in Haemaphysalis ticks and wild animals in the western part of Japan
Source: PLoS One. 2017 Mar 31;12(3):e0174727. doi: 10.1371/journal.pone.0174727 (PMC5375152; doi:10.1371/journal.pone.0174727)
Supplement: S2 Table — (DOCX) [file pone.0174727.s002.docx]

**S2 Table. Primer list**

| Gene | Name | Sequences (5’-3’) | References |
| --- | --- | --- | --- |
| *clpA* | HTRF_clpA_1292F | TCTTGGAGCTAAATTTAAGCTTG | In this study |
|  | RF_clpA_R | TTAAYAAAAYTATTCAYCTCTTC | 26 |
| *clpX* | RF_clpX_243F | ATATTATTGGRCARGAAGATGC |  |
|  | HTRF_clpX_1318R | CAATTGATTTCATAAAGCTCTTTTGCC | In this study |
| *nifS* | HTRF_nifS_9018F | CCAAATTCTCTAGCCTGTAAAC |  |
|  | HTRF_nifS_792R | CCAATTCCTGTTGGAGCAAGCAT |  |
| *pepX* | HTRF_pepX_12125F | CTCCCCCAAGAGTTGTYACAAT |  |
|  | HTRF_pepX_773R | GAGTGCATTCCCCACATTG |  |
| *pyrG* | RF_pyrG_F | TTGGTGGTACTGTRGGRGATATGG | 26 |
|  | RF_pyrG_R | AAGTTGCATKCCAAGACAAATRCCAAG |  |
| *recG* | HTRF_recG_9245F | GATCAAGAAGTTGCAATTGATG | In this study |
|  | HTRF_recG_10489R | GTCCAAAACGCTCAGCAT |  |
| *rplB* | RF_rplB_40F | TCTTTRCGTTATAAGACAAC | 26 |
|  | HTRF_rplB_1077R | GCTGACCCCAAGGAGATACAGG | In this study |
| *uvrA* | HTRF_uvrA_2850F | GAACATAATAGTTGTCCTTTGTGTG |  |
|  | uvrA_2235R_RF | TAACATTAAAAGAAAAYCTACC | 26 |
| *16S rDNA* qPCR | 16S RT-F | GCTGTAAACGATGCACACTTGGT | 27 |
|  | 16S RT-R | GGCGGCACACTTAACACGTTAG |  |
|  | BS-16S | FAM-CGGTACTAATCTTTCGATTA-MGB | 15 |
| *16S rDNA* conventional PCR | rrs-F1 3-26 | ATAACGAAGAGTTTGATCCTGGCT | 2 |
|  | rrs-R4 1542-1520 | AAAGGAGGTGATCCAGCCRCACT |  |
|  | rrs-F2(682-703)^*1^ | GGTGTAAGGGTGGAATCTGTTG |  |
|  | rrs-R3(749-768) ^*1^ | TTTCGTGACTCAGCGTCAGT |  |
|  | 14-XbaR2^*1^ | GTCCCGCAACGAGCGCAACC |  |
| *flaB* | BflaPAD | GATCARGCWCAAYATAACCAWATGCA | 2 |
|  | BflaPDU | AGATTCAAGTCTGTTTTGGAAAGC |  |
|  | BflaPBU | GCTGAAGAGCTTGGAATGCAACC |  |
|  | BflaPCR | TGATCAGTTATCATTCTAATAGCA |  |
| *glpQ* | glpQ F | GGTATGCTTATTGGTCTTC | 28 |
|  | glpQ R | TTGTATCCTCTTGTAATTG |  |

*1 The primer used for sequencing.
