# Supplementary material for: Epidemiological study of relapsing fever borreliae detected in Haemaphysalis ticks and wild animals in the western part of Japan
Source: PLoS One. 2017 Mar 31;12(3):e0174727. doi: 10.1371/journal.pone.0174727 (PMC5375152; doi:10.1371/journal.pone.0174727)
Supplement: S3 Table — (DOCX) [file pone.0174727.s003.docx]

**S3 Table. The bacterial genotype and tick species positive for *Borrelia* spp. in this study**

| ID | Region | Tick species | Tick stage | Number of ticks/ pool | Genotype |
| --- | --- | --- | --- | --- | --- |
| W-21 | Wakayama | *Haemaphysalis flava* | Male | 4 | HF |
| W-31 | Wakayama | *Haemaphysalis kitaokai* | Female | 5 | HK |
| W-88 | Wakayama | *Haemaphysalis megaspinosa* | Nymph | 20 | HM |
| W-126 | Wakayama | *Haemaphysalis flava* | Female | 4 | HF |
| W-130 | Wakayama | *Haemaphysalis megaspinosa* | Nymph | 20 | HM |
| W-147 | Wakayama | *Haemaphysalis formosensis* | Nymph | 9 | HF |
| W-174 | Wakayama | *Haemaphysalis flava* | Male | 5 | HF |
| W-175 | Wakayama | *Haemaphysalis flava* | Male | 4 | HF |
| W-188 | Wakayama | *Haemaphysalis flava* | Nymph | 17 | HF |
| W-282 | Wakayama | *Haemaphysalis flava* | Nymph | 13 | HF |
| W-294 | Wakayama | *Haemaphysalis longicornis* | Nymph | 20 | HF |
| W-321 | Wakayama | *Haemaphysalis longicornis* | Male | 5 | HL |
| W-460 | Wakayama | *Haemaphysalis megaspinosa* | Nymph | 13 | HM |
| W-473 | Wakayama | *Haemaphysalis megaspinosa* | Nymph | 16 | HM |
| W-478 | Wakayama | *Haemaphysalis flava* | Nymph | 14 | HF |
| W-493 | Wakayama | *Haemaphysalis megaspinosa* | Nymph | 20 | HM |
| W-501 | Wakayama | *Haemaphysalis flava* | Male | 5 | HF |
| W-509 | Wakayama | *Haemaphysalis flava* | Nymph | 20 | HF |
| W-522 | Wakayama | *Haemaphysalis megaspinosa* | Nymph | 20 | HM |
| W-555 | Wakayama | *Haemaphysalis longicornis* | Nymph | 20 | HL |
| W-557 | Wakayama | *Haemaphysalis longicornis* | Nymph | 20 | HL |
| 16 | Shimonoseki | *Haemaphysalis megaspinosa* | Female | 1 | HM |
| 191 | Shimonoseki | *Haemaphysalis megaspinosa* | Male | 1 | HF |
| L-29 | Shimonoseki | *Haemaphysalis kitaokai* | Larva | 2 | HK |
