# Supplementary material for: Epidemiological study of relapsing fever borreliae detected in Haemaphysalis ticks and wild animals in the western part of Japan
Source: PLoS One. 2017 Mar 31;12(3):e0174727. doi: 10.1371/journal.pone.0174727 (PMC5375152; doi:10.1371/journal.pone.0174727)
Supplement: S4 Table — (DOCX) [file pone.0174727.s004.docx]

**S4 Table. Genetic group mean pairwise distance for *flaB* among 4 types of *Borrelia* spp. in this study.**

|  | *Borrelia* sp. HF | *Borrelia* sp. HK | *Borrelia* sp. HM | *Borrelia* sp. HL |
| --- | --- | --- | --- | --- |
| *Borrelia* sp. HF |  |  |  |  |
| *Borrelia* sp. HK | 99.1% |  |  |  |
| *Borrelia* sp. HM | 99.5% | 99.1% |  |  |
| *Borrelia* sp. HL | 95.0% | 95.3% | 95.5% |  |
