# Supplementary material for: Epidemiological study of relapsing fever borreliae detected in Haemaphysalis ticks and wild animals in the western part of Japan
Source: PLoS One. 2017 Mar 31;12(3):e0174727. doi: 10.1371/journal.pone.0174727 (PMC5375152; doi:10.1371/journal.pone.0174727)
Supplement: S5 Table — (DOCX) [file pone.0174727.s005.docx]

**S5 Table. Genetic group mean distance of *16S rDNA* (right upper) or *glpQ* (left lower) of *Borrelia* spp. in this study and other hard-bodied tick-borne relapsing fever borreliae.**

|  | *Borrelia* sp. | *B. lonestari* | *B. theileri* |
| --- | --- | --- | --- |
| *Borrelia* sp. | . | 98.8％ | 99.4％ |
| *B. lonestari* | 92.7% |  | 99.0％ |
| *B. theileri* | 92.5% | 92.0% |  |
