# Supplementary material for: Epidemiological study of relapsing fever borreliae detected in Haemaphysalis ticks and wild animals in the western part of Japan
Source: PLoS One. 2017 Mar 31;12(3):e0174727. doi: 10.1371/journal.pone.0174727 (PMC5375152; doi:10.1371/journal.pone.0174727)
Supplement: S6 Table — (DOCX) [file pone.0174727.s006.docx]

**S6 Table. Prevalence of *Borrelia* sp. in wild raccoons collected from Wakayama Prefecture**

| Month | Number of samples | Number of positive samples (prevalence %) | | | | | |
| --- | --- | --- | --- | --- | --- | --- | --- |
|  |  | *Borrelia* sp. HF | *Borrelia* sp. HK | *Borrelia* sp. HM | *Borrelia* sp. HL | Co-infection | Total |
| Jan | 10 | 0 | 0 | 0 | 0 | 0 | 0 |
| Feb | 10 | 0 | 0 | 0 | 0 | 0 | 0 |
| Mar | 10 | 0 | 0 | 0 | 0 | 0 | 0 |
| Apr | 10 | 0 | 0 | 0 | 0 | 0 | 0 |
| May | 10 | 0 | 0 | 0 | 0 | 0 | 0 |
| Jun | 10 | 0 | 0 | 0 | 0 | 0 | 0 |
| Jul | 10 | 0 | 0 | 0 | 0 | 0 | 0 |
| Aug | 10 | 1 | 0 | 0 | 0 | 0 | 1 (10%) |
| Sep | 10 | 0 | 0 | 0 | 0 | 0 | 0 |
| Oct | 10 | 0 | 0 | 0 | 0 | 0 | 0 |
| Nov | 10 | 0 | 0 | 0 | 0 | 0 | 0 |
| Dec | 10 | 0 | 0 | 0 | 0 | 0 | 0 |
| Total | 120 | 1 (0.83%) | 0 | 0 | 0 | 0 | 1 (0.83%) |
