# Supplementary material for: Epidemiological study of relapsing fever borreliae detected in Haemaphysalis ticks and wild animals in the western part of Japan
Source: PLoS One. 2017 Mar 31;12(3):e0174727. doi: 10.1371/journal.pone.0174727 (PMC5375152; doi:10.1371/journal.pone.0174727)
Supplement: S1 Fig — (DOCX) [file pone.0174727.s007.docx]

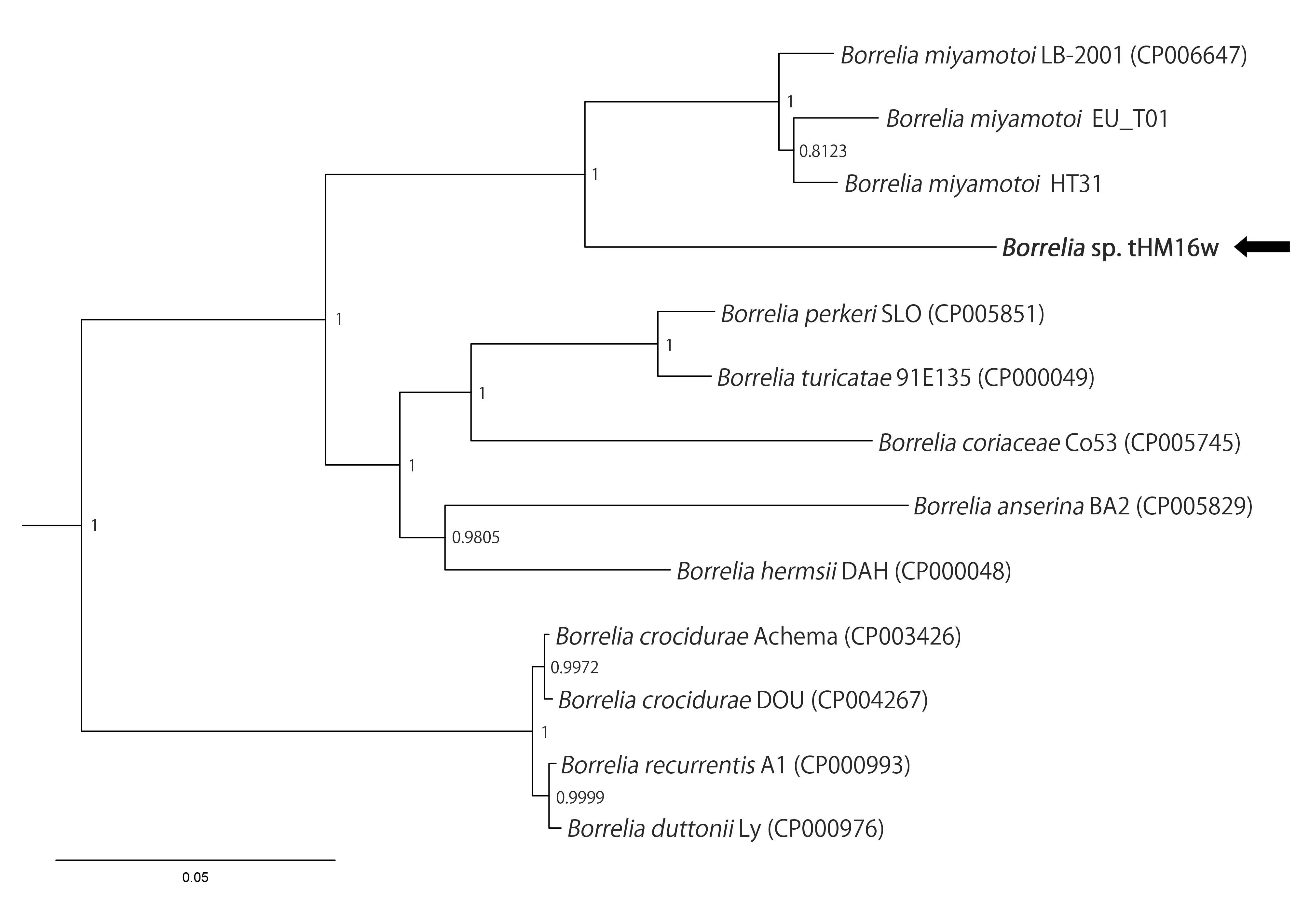


**S1 Fig. Bayesian phylogenetic analysis of borrelial housekeeping gene sequences.**This phylogenetic tree was constructed by Bayesian phylogenetic analysis as described previously by Margos et al. (2008). Bayesian Posterior Probabilities of clades were provided. Scale bar (0.05) indicates 5% divergence. The LD borreliae (ST1 [*B. burgdorferi* B31], ST84 [*B. garinii*], and ST70 [*B. afzeli* VS461]) and ST633 (*B. miyamotoi* HT31), and ST635 (*B. miyamotoi* EU_T01) were downloaded from the MLST website; www.mlst.net. LD borreliae were used as outgroups (data not indicated). Pointing arrows and bold type indicate the results obtained in the present study. Numbers in parentheses represent GenBank accession numbers or ST numbers on the MLST website.
